# A study on Chinese ancient jades with mercury alteration unearthed from Lizhou’ao Tomb

**DOI:** 10.1038/s41598-019-55138-2

**Published:** 2019-12-27

**Authors:** Yi Bao, Changqing Xu, Qinwen Zhu, Yuesheng Li

**Affiliations:** 1 Department of Materials Science, Fudan University, Shanghai, 200433 P.R. China; 2 Institute of Archaeological Science, Fudan University, Shanghai, 200433 P.R. China; 3 China University of Geosciences (Wuhan), Wuhan, 430074 P.R. China; 4 Jiangxi Provincial Institute of Cultural Relics and Archaeology, Nanchang, 330008 P.R. China

**Keywords:** Solid Earth sciences, Materials science

## Abstract

“Alteration” geologically refers to chemical composition and/or structural changes of minerals under the influences of hydrothermal fluids, surface water, seawater, or other environmental conditions. In this paper, we use the word “alteration” to refer to chemical component and structural changes in jade artifacts caused by human activity and natural weathering, which is different from the term in geology. “Mercury alteration”, a kind of black alteration related to Hg, is unique among the several types of alteration that occur in Chinese ancient jades. Mercury alteration often appears on ancient jade artifacts unearthed from high-grade tombs of the pre-Qin period (before 221 B.C.). Therefore, ancient jades with mercury alteration have attracted substantial attention from Chinese archaeologists. This paper reports the use of materials analytic techniques to study such ancient jade fragments. The studied jade samples date to the middle and late periods of the Spring and Autumn Period (~500 B.C.) and were unearthed from Lizhou’ao Tomb in Jiangxi Province, China. Structural analyses revealed the internal microstructure of the ancient jade fragments and the microdistribution of the mercury alteration. The jade fragments exhibit typical characteristics of round holes and structural hierarchy, which imply that the jades were heated before burial. The black alteration on these jade samples was found to be rich in Hg. The results of this study will be widely useful in the study of ancient jade artifacts and jade culture in Chinese archeology.

## Introduction

As one of the most characteristic artifacts in China, ancient jade is a significant representative of traditional Chinese culture. Chinese jade culture has lasted more than 8000 years without interruption^1^. Jade not only served as a decoration in ancient Chinese life but also symbolized status and power in Chinese culture. An important typical characteristic of ancient jade artifacts from archaeological excavation is also called “alteration”^2–4^. The term “alteration” here is referred to chemical component and structural changes in jade artifacts caused by human activity and natural weathering, which is different from the term in geology as described above^5^. The alteration of ancient jades has been categorized according to 7 colors: black, white, yellow, red, blue, green and purple. Each color is produced by different mechanism^6^. A particular type of black alteration is “mercury alteration”, which is related to Hg^7^. Mercury alteration is typically found on ancient jades unearthed from tombs of high-class nobles. Therefore, the study of ancient jades with mercury alteration is important for Chinese archeology.

Mercury alteration is recorded in ancient Chinese texts. Xu^8^, Chen^9^ and Liu^10^ considered that a kind of black alteration on unearthed jades was related to mercury. This kind of black alteration was termed “Shuiyin qin” or “Heiqigu”. The word “Shuiyin qin” was first used to refer to a kind of copper mirror with a white surface dating from the Eastern Han Dynasty (25 A.D.–220 A.D.), and the word “Heiqigu” was first used in connection with a kind of copper mirror with a black surface dating from the Tang Dynasty (618 A.D.–907 A.D.)^7^. “Shuiyin qin” and “Heiqigu” can mean different things when used to refer to different materials (Table 1). In terms of color, “Shuiyin qin” and “Heiqigu” in jade artifacts are both black. “Shuiyin qin” on a copper mirror is white, while “Heiqigu” on a copper mirror is black. In terms of material, “Shuiyin qin” and “Heiqigu” in jades both refer to alteration relative to mercury, while “Shuiyin qin” and “Heiqigu” on copper mirrors both relate to tin. In terms of the shape of the black alteration, according to custom, “Shuiyin qin” refers to black alteration in the shape of lines or small spots, while “Heiqigu” refers to black alteration that covers all or half of a jade artifact^7^.

**Table 1:** Proper names of mercury alteration in Chinese ancient literatures.

Name	Object	Color	Factor	Shape	Start using time
Shuiyin Qin	Ancient jades	Black	Hg	Line or spot	
	Ancient copper mirror	White	Sn	On surface	Eastern Han Dynasty
Heiqigu	Ancient jades	Black	Hg	Part or whole	
	Ancient copper mirror	black	Sn	On surface	Tang Dynasty

Ancient jade artifacts with mercury alteration from 5 tombs of the pre-Qin period (before 221 B.C.) have been examined using X-ray fluorescence (XRF): Fuhao Tomb (in press), Yejiashan Tomb, Yuehe Tomb^11,12^, Jiuli Tomb^12^ and Yangjiashan Tomb^7,13^ (Table 2). The ancient jades with mercury alteration unearthed from these tombs have some common characteristics. In terms of time period, most date to the period between the Shang Dynasty and the Warring States Period (1600 B.C.–221 B.C.). This period was important to the formation of the Chinese nation. Geographically, such jade artifacts are mainly distributed along the middle reaches of the Yangtze River and the middle reaches of the Yellow River, which together represent the core area of the Chinese nation during that period. According to the tomb degree system used in archeology, these jades were mainly unearthed from high-grade tombs, particularly tombs from the Shang Dynasty and the Western Zhou Dynasty. The research samples described in this study are ancient jade artifacts with mercury alteration unearthed from Lizhou’ao Tomb. The results of this paper indicate a widening of the distribution of ancient jades with mercury alteration to Jiangxi Province and a richer variety of samples than previously assumed (Table 2).

**Table 2:** The unearthed ancient jade artifacts identified with mercury alteration.

Tomb	Fuhao	Yejiashan	Yuhe	Lizhou’ao	Jiuli	Yangjiashan
Time	Shang Dynasty (1600 B.C.–1046 B.C.)	Western Zhou Dynasty (1046 B.C.–771 B.C.)	Spring and Autumn Period (770 B.C.–476 B.C.)	Spring and Autumn Period (770 B.C.–476 B.C.)	Warring States Period (475 B.C.–221 B.C.)	Warring States Period (475 B.C.–221 B.C.)
Province	Henan Province	Hubei Province	Henan Province	Jiangxi Province	Hunan Province	Hunan Province
Location	Middle of the Yellow River	Middle of the Yangtze River	Middle of the Yellow River	Middle of the Yangtze River	Middle of the Yangtze River	Middle of the Yangtze River
Status of Owner	Queen	Vassal King	Vassal King	Noble	Vassal King	Noble
Tomb Grade	High	High	High	High/Middle	High	Low
References	In press	Luo *et al*. 2018	Xu and Wang^12^	This paper	Zhao *et al*.^13^	Zhao *et al*.^13^

Owing to the age and degree of these tombs, the study of the ancient jade artifacts with mercury alteration found in them has attracted considerable archaeological research attention. A small number of ancient jades with mercury alteration were previously examined by XRF^12–14^. The results agreed with the findings of ancient Chinese texts. Xu^8^, Chen^9^ and Liu^10^ considered that a kind of black alteration appearing on unearthed jades was related to mercury and referred to it as “Shuiyin qin” and “Heiqigu” in Chinese. Wen studied Chinese ancient jade materials using a geological method systematically combined with the findings of the ancient Chinese literature^15–17^. Gaines conducted the first study on mineralogical alteration in Chinese ancient jades using X-ray diffraction (XRD) and scanning electron microscopy (SEM)^18^. Douglas and Wen proposed that certain Chinese ancient jades might have been heated during processing and during cremation in the historical period mainly using XRD and Fourier transform infrared spectroscopy (FTIR)^19^. Preliminary heating experiments by Bao using several advanced characterization methods, including XRD, a thermogravimetric analyzer (TGA), an ultraviolet-visible spectrophotometer (UV-Vis), FTIR and SEM equipped with energy-dispersive spectroscopy (SEM-EDS), revealed appearance changes in nephrite jade that occurred during the heating process^20^.

**Figure 1:**
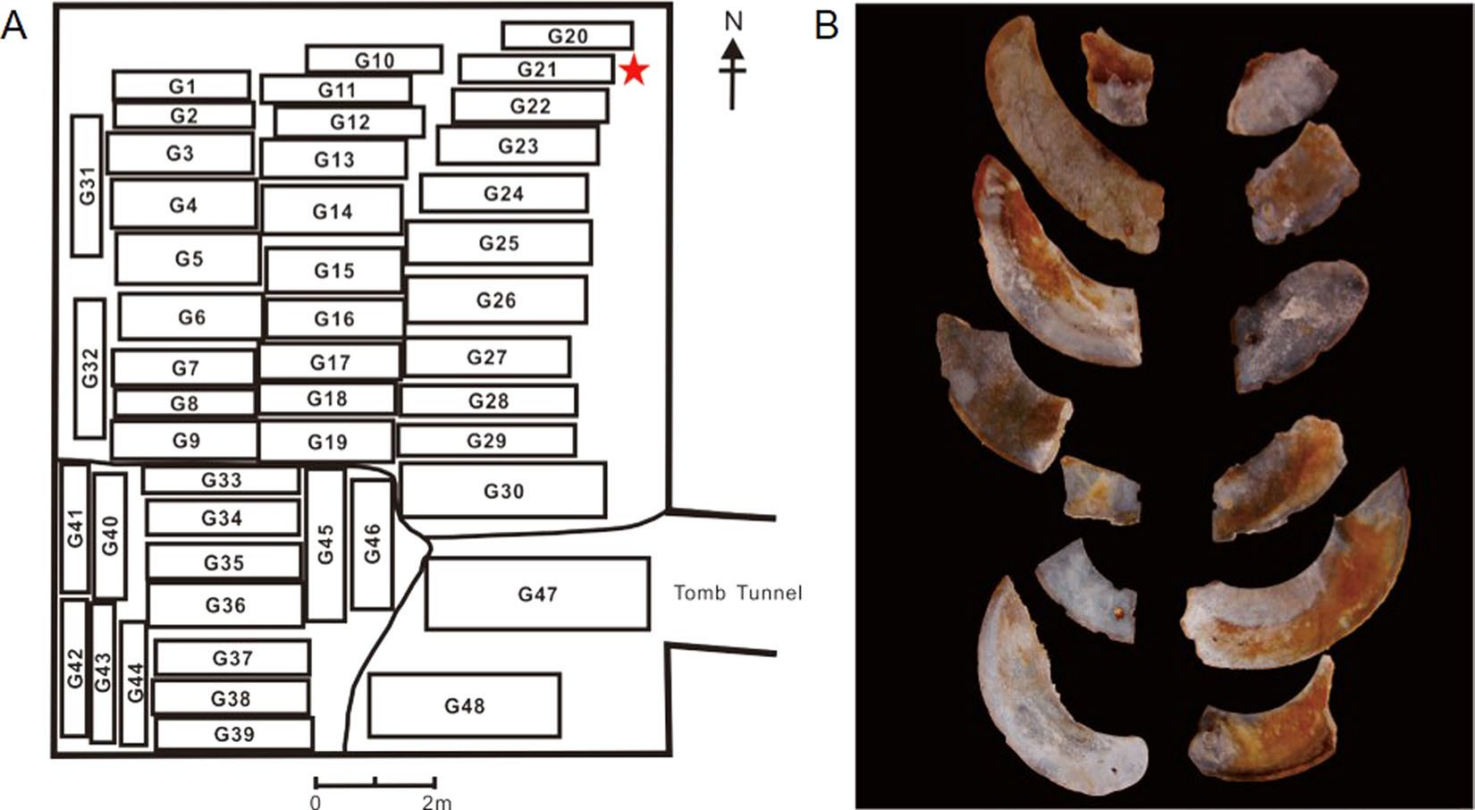
Archaeological data of the Lizhou’ao Tomb. (**A**) Diagram of burial coffins in the Lizhou’ao Tomb. Star labels the location of G21. (**B**) The photo of all the ancient jades unearthed from G21.

The ancient jade fragment samples studied in this paper were unearthed from Lizhou’ao Tomb and date to the middle and late period of the Spring and Autumn Period (~500 B.C.). Lizhou’ao Tomb was excavated in Jing’an, Jiangxi Province, in 2007. This tomb is famous for its extraordinary structure, which is a single-pit multicoffin grave (Fig. 1A). This grave is widely known as the earliest tomb found in China and the one with the greatest number of coffins^21,22^. Jiangxi Province was the transition zone of the Han nation and the Yue nation during the middle and late period of the Spring and Autumn Period. Certain researchers believe that Lizhou’ao Tomb reflects the fusion of the two nations^23^. This special tomb reflects both the diversity and the unity of Chinese culture. A total of 11 pieces of ancient jade have been unearthed from the tomb, 9 of which belong to G21 (Fig. 1B).

## Materials and Methods

### Description of ancient jade samples

**Figure 2:**
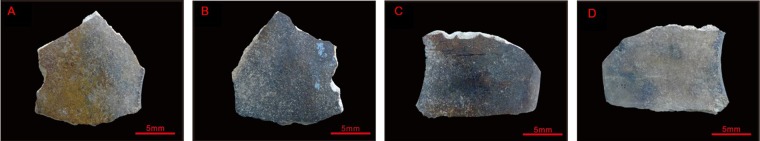
The photos of G21:2-1 and G21:2-2. (**A**,**B**) Are G21:2-1, (**C**,**D**) are G21:2-2.

Ancient jade fragments unearthed from G21 of Lizhou’ao Tomb were studied in this paper (Fig. 1B). The jade samples were provided by Jiangxi Provincial Institute of Cultural Relics and Archaeology^24^. All 9 pieces of ancient jade unearthed from G21 have similar characteristics. G21:2-1 and G21:2-2, which exhibit the best phenomenological features, were chosen as representative study samples. Details of the two samples are listed in a table that presents the ancient jade analysis system (Table 3). G21:2-1 and G21:2-2 both display three kinds of color: black, yellowish-brown and white (Fig. 2). These three colors are uncommon in ancient jades, which usually white to green.

**Table 3:** The ancient jade system research data of G21:2-1 and G21:2-2.

	Sample	Basic characteristics			Alteration characteristics			Jade characteristics	
		Color	Glossiness	Transparency	Color	Distribution	Degree	Color	Structure
1	G21:2-1	One side is yellowish-brown, another side is black, the inner part is white.	Earthy luster	Opacity	Black, yellowish-brown, white	Face shape and body shape	Hard	Unknown	Compact
2	G21:2-1	One side is yellowish-brown, another side is mainly black, has some white and yellowish-brown, inner part is white	Earthy luster	Opacity	Black, yellowish-brown, white	Face shape and body shape	Hard	Unknown	Compact

### Analytic techniques used in this study

Comprehensive visual, compositional and microstructural nondestructive examinations were employed. The phase compositions of the ancient jade samples were analyzed using XRD (D8 ADVANCE). FTIR (Nicolet 6700, Thermo Fisher, USA) was employed to analyze the OH^−^ of the tremolite-actinolite in the samples. UV-Vis (Lambda 750, PerkinElmer, USA) was used to record the visible absorption spectra of the samples in the range of 400–800 nm. SEM-EDS (Phenom ProX, Phenom-World, Netherlands) was used to capture the microstructure images and to probe the compositions of the jade samples. X-ray computed tomography (CT) (Phoenix v|tome|x m, General Electric Company, USA), a nondestructive method, was employed to visualize the inner structure of the samples.

## Results and Discussion

### Analytic result for the material of the ancient jade artifacts

**Figure 3:**
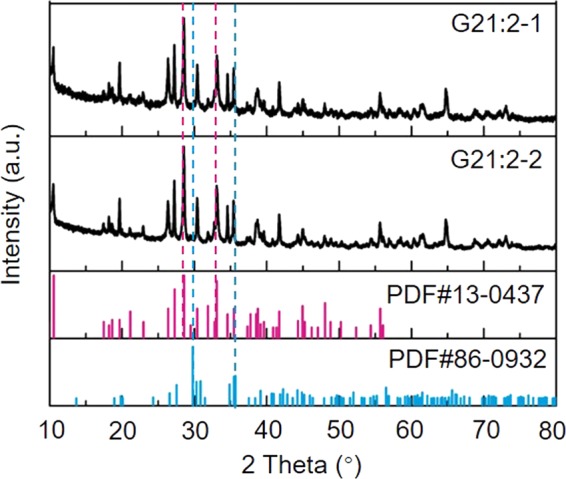
The XRD data of G21:2-1 and G21:2-2. PDF# 13-0437 and PDF# 86-0932 are representative XRD patterns of tremolite and diopside, respectively.

The phase identification of the two ancient samples from Lizhou’ao Tomb is necessary since their appearances are unlike those of normal jades. XRD was employed to investigate the mineral phase of the two samples. Figure 3 shows the XRD results. The samples are nearly pure tremolite, with tremolite proportions over 95%. In the pattern of G21:2-1, the characteristic diffraction peaks appear at 2θ = 28.52°, 10.54°, 27.20° for tremolite. In the pattern of G21:2-2, the characteristic diffraction peaks appear at 2θ = 28.52°, 10.46°, 27.20° for tremolite. The spectrograms of both samples conform to the spectrum of a standard tremolite sample.

The main mineral of nephrite is the series tremolite-actinolite [Ca_2_(Mg, Fe)_5_Si_8_O_22_(OH)_2_]. Nephrite consists of two minerals of the amphibole group: tremolite and actinolite. The two minerals are distinguished by the ratio of magnesium and iron in their chemical composition. A mineral is tremolite when *f * = *Mg/*(*Mg* + *Fe*) ≥ 0.9 and actinolite when *f* is 0.5–0.9^25^. According to the EDS data (Table 4), the *f* values of G21:2-1 and G21:2-2 are ~0.96 and ~0.95, respectively. Therefore, G21:2-1 and G21:2-2 are both tremolite nephrite jade.

**Table 4:** The EDS data of G21:2-1 and G21:2-2. *f* = *Mg/*(*Mg* + *Fe*).

Sample	Element Symbol	O	Si	Mg	Ca	Al	Fe	Hg	*f*
G21:2-1	Atomic Conc. (%)	63.7	18.8	13.5	3.0	0.6	0.5	0.0	~0.96
G21:2-2	Atomic Conc. (%)	61.5	20.2	13.2	4.0	0.7	0.5	0.0	~0.95

### Evidence for the heat treatment of the ancient jade artifacts

Although the two studied samples are nephrite jades, they do not exhibit the white to green color typical of such jades (Fig. 2). The color features of the two samples resemble those of heated nephrite jades, indicating a heating treatment.

**Figure 4:**
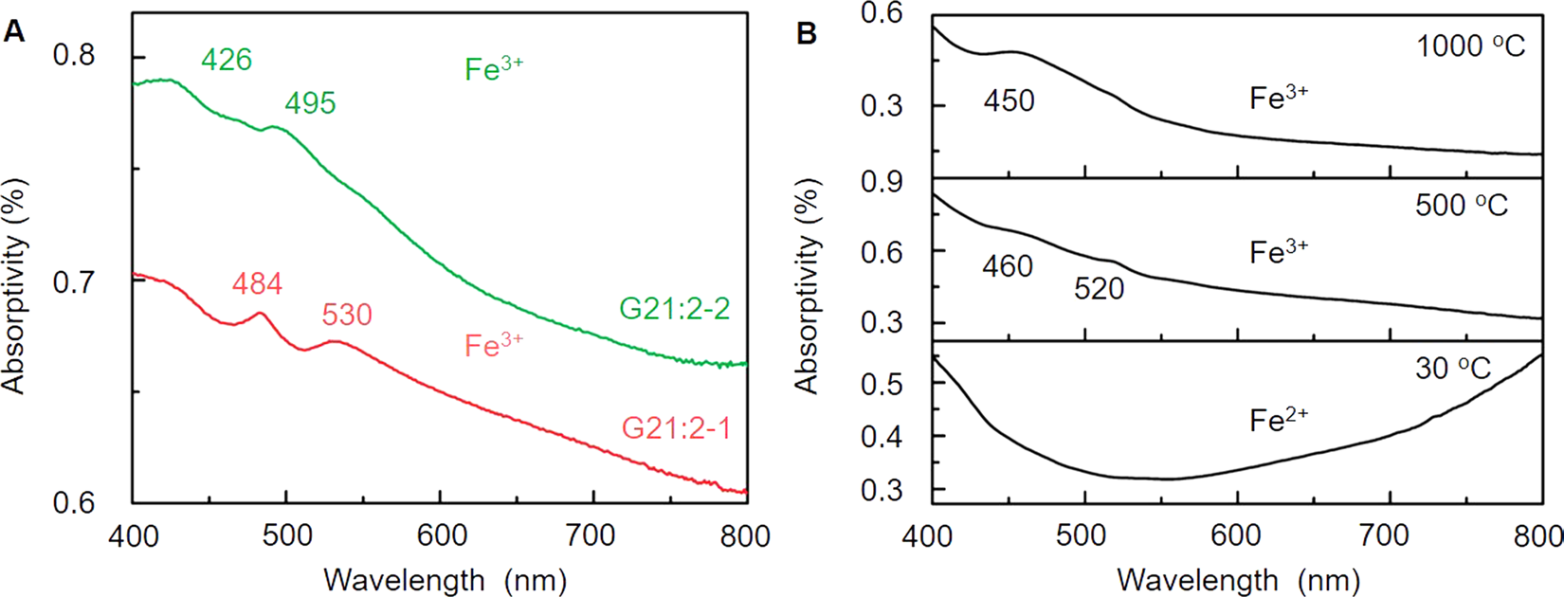
The UV-Vis data of G21:2-1 and G21:2-2 (**A**) and heated nephrite jades (**B**)^20^.

The UV-Vis data indicate that the yellowish-brown color of the two ancient jade fragments is due to the coloring element Fe^3+^. In Fig. 4A, G21:2-1 has 484 nm and 530 nm peaks, and G21:2-2 has 426 nm and 495 nm peaks. These UV-Vis peaks all belong to coloring element Fe^3+^. Fe^3+^ mainly produces a yellowish-brown color, which is due to the ligand-to-metal charge transfer (LMCT) for *O*^2−^ → *Fe*^3+^. The main coloring element of unheated nephrite jades is Fe^2+^, which produces a green color because of the coupled pairs *Fe*^*2+*^  → *Fe*^3+^ and *Fe*^*2+*^(^5^*T*_2_) + *Fe*^*3+*^(^6^*A*_1_) → *Fe*^*2+*^(^5^*E*) + *Fe*^*3+*^(^6^*A*_1_) (Fig. 4B)^26^. According to a previous study, Fe^2+^ oxidizes to Fe^3+^ during the heating process in the temperature range of 500–600 °C (Fig. 4B). Fe^3+^ becomes the main coloring element of jade when the heating temperature surpasses 500 °C (Fig. 4B)^20^. The yellowish-brown produced by Fe^3+^ is the representative color and a diagnostic attribute of heated nephrite jades. The UV-Vis data for G21:2-1 and G21:2-2 indicate these two ancient jade artifacts were heated over 500 °C in the distant past.

**Figure 5:**
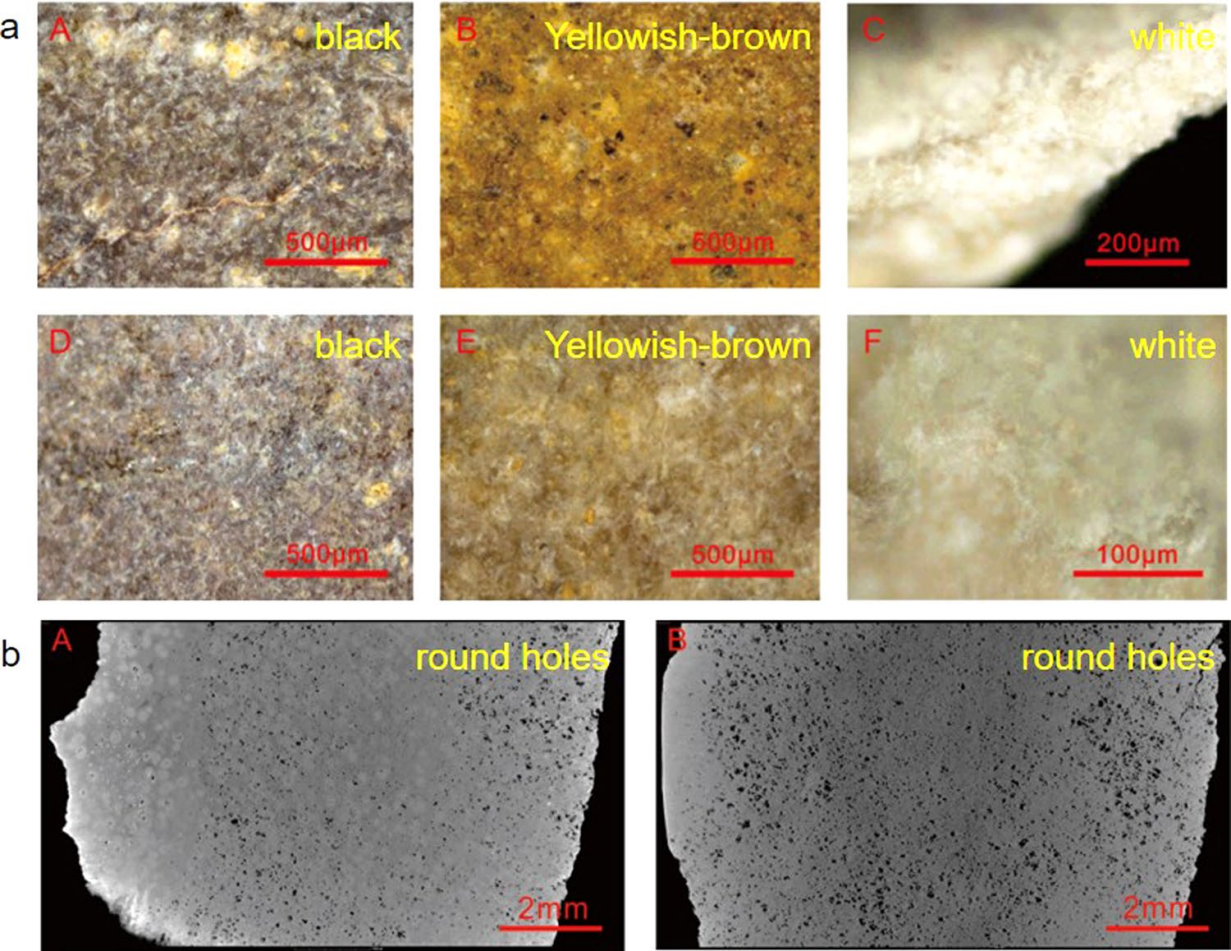
The micro photos and CT images of G212-1 and G21:2-2. (**a**) The micro photos. (**A**,**B**,**C**) the micro photos of G212-1. (**D**,**E**,**F**) the micro photos of G212-2. (**b**) The CT images of G212-1.

Heated tremolite nephrite jades exhibit the characteristic color hierarchy feature of white inside and yellowish-brown outside when the heating temperature surpasses 500 °C according to the previously cited study^20^. The two studied jade fragments can be assigned to outside and inside sections according to their color, as shown in the microphotos in Fig. 5a. The outside sections have black and yellowish-brown colors. The inside sections are white. This phenomenon is referred to as the color hierarchy. Therefore, these two ancient jades were heated over 500 °C in historical time. The heating process mainly produced yellowish-brown and white on the ancient jades. The black color was produced by materials from the external environment.

Heated tremolite nephrite jades exhibit the diagnostic structural feature of round holes, and this feature also indicates a heating temperature over 800 °C, based on the previously cited study^20^. The CT image in Fig. 5b shows the microstructure of the ancient jade fragments. These two ancient jade samples exhibit obvious round holes. Therefore, the two jade samples were heated over 800 °C.

**Figure 6:**
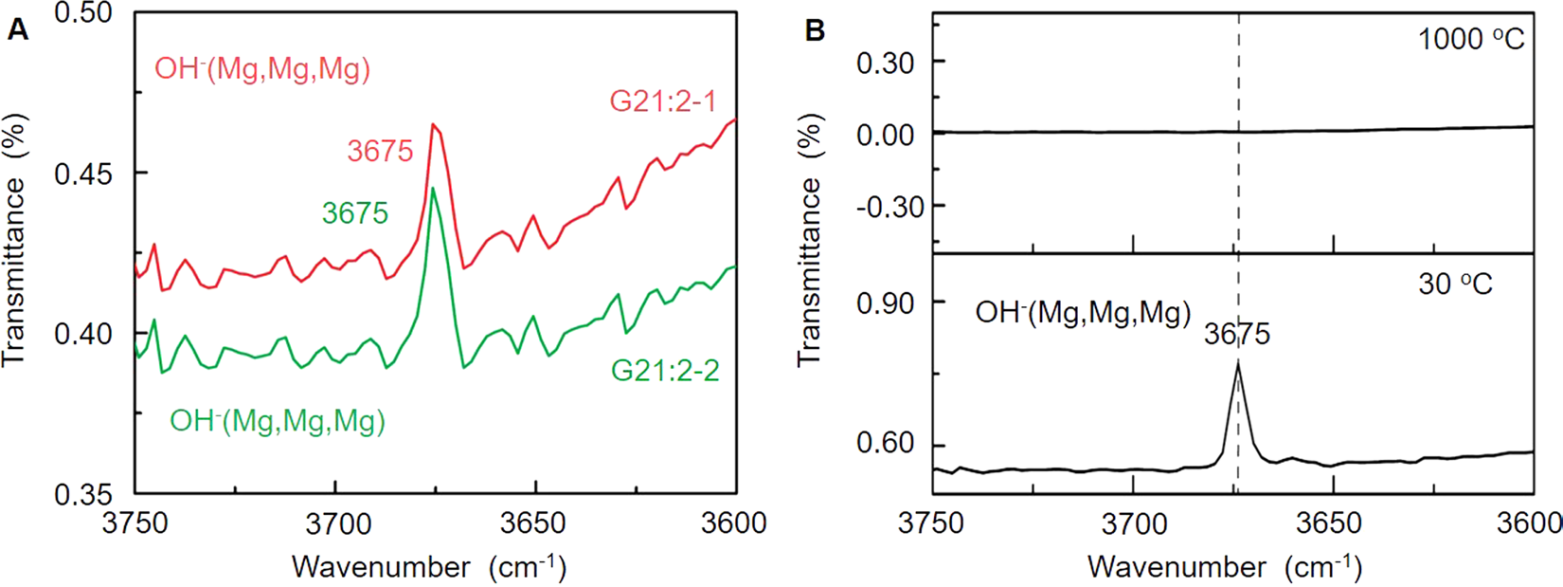
The FTIR data of G21:2-1 and G21:2-2 (**A**) and heated nephrite jades (**B**)^20^.

According to the previously cited study, tremolite in nephrite jades loses OH^−^ and transforms to diopside, enstatite, SiO_2_ and H_2_O (gas) when the heating temperature exceeds 1000 °C (Fig. 6B)^20^. The FTIR data indicate that the two ancient jade fragments have values of 3675 cm^−1^, which belongs to OH^−^ (Mg, Mg, Mg) (Fig. 6A). The XRD data in Fig. 3 indicate the two jades are nearly pure tremolite and contain no diopside. As a result, these two nephrite jades did not lose OH^−^ or transform. Therefore, it is highly likely these two nephrite jades were not heated over 1000 °C.

In sum, one can conclude that these two ancient nephrite jade fragments unearthed from Lizhou’ao Tomb were heated to the temperature range of 800–1000 °C in ancient times.

### Material of the mercury alteration on the ancient jade artifacts

According to the preceding analysis, the black color originates in the external environment. Therefore, the black color represents a kind of black alteration on the studied ancient jades.

**Figure 7:**
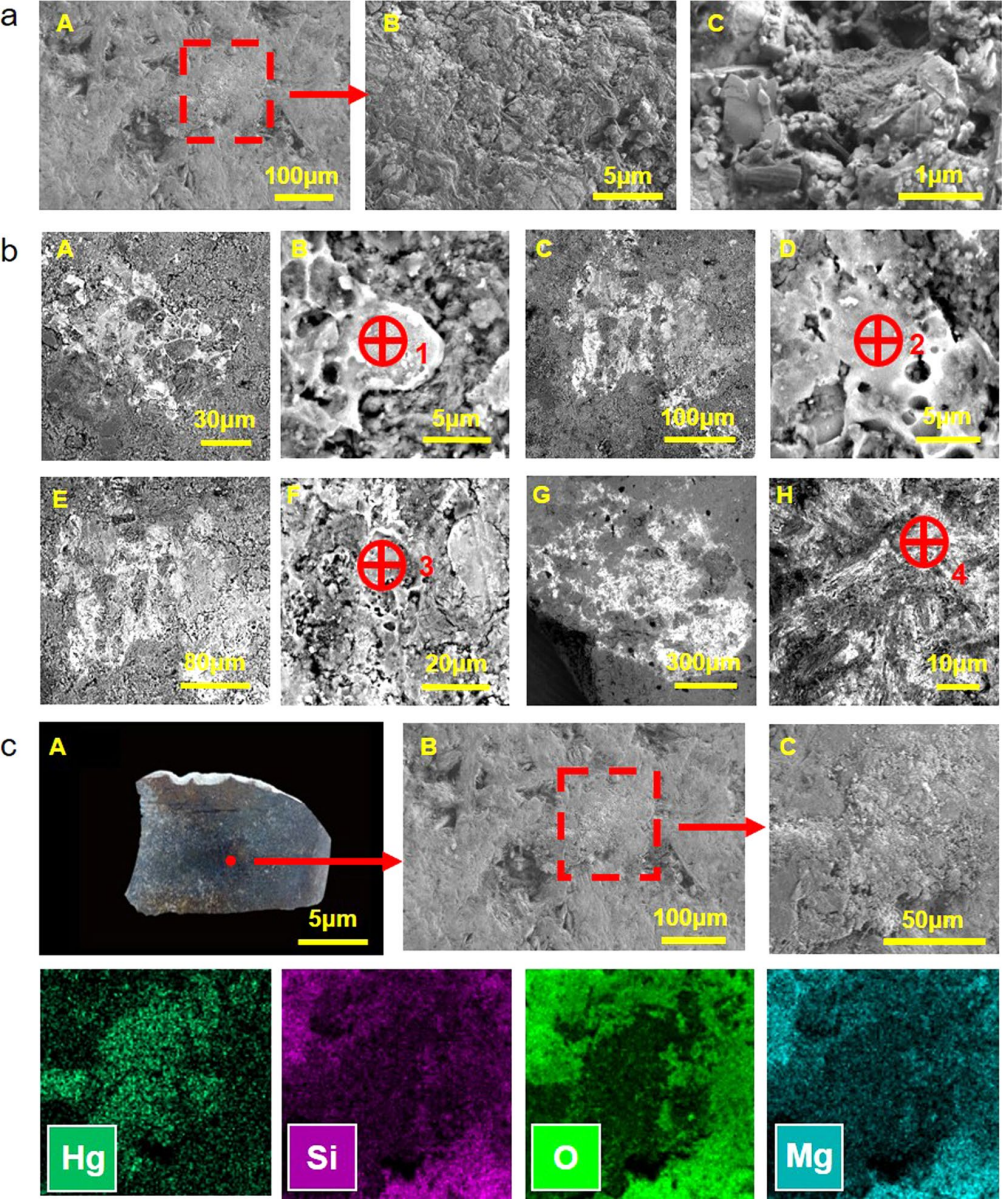
The SEM image and the corresponding elemental mapping of the mercury alteration of G212-1 and G21:2-2. (**a**) The secondary electron images of G212-1. (**b**) The backscattered electron images of G21:2-2. The 4 sites in images were chosen to take EDS data. (**c**) The corresponding elemental mapping of the mercury alteration on G212-1.

The SEM image in Fig. 7a,b shows that the black color of the studied jades is distributed on the surface of the samples and does not belong to the jade material itself. Four sites on the two jade samples were chosen from which to take EDS data (Fig. 7b). The values are listed in Table 5. The atomic concentrations of Hg at the four sites are 91.6 at%, 83.0 at%, 49.0 at% and 34.4 at%, respectively. Note that the black alteration contains a high amount of Hg, up to 91.6 at%. These data indicate that Hg is one of the main elements of the black alteration.

**Table 5:** The EDS data of the mercury alteration on G21:2-1 and G21:2-2. Site 1 and 2 are on G21:2-1 (Fig. 2B). Site 3 and 4 are on G21:2-2 (Fig. 2D). The EDS sites are shown in Fig. 7b.

Element Symbol	Atomic Conc. (%)			
	Site 1	Site 2	Site 3	Site 4
Hg	91.6	83.0	49.0	34.4
O	4.1	5.3	26.5	36.5
N	1.2	3.2	7.9	10.5
C	0.6	5.3	6.1	4.5
S	0.7	1.6	0.7	0.4
Si	0.7	0.5	3.0	5.9
Fe	0.6	0.3	3.1	2.0
Mg	0.2	0.2	1.0	3.3
Ca	0.2	0.1	0.8	1.8
Al	0.1	0.4	1.3	0.4
P	0.1	0.1	0.2	0.1
K	\	\	0.2	\
Cl	\	\	0.1	\
As	0.1	\	\	\

An elemental map of the black color is shown in Fig. 7c. Si, O and Mg are the main elements of tremolite in nephrite jade. Hg belongs to the black color. The different distribution phenomena of the jade material and the alteration represent further proof that the black color is an alteration on the ancient jade. The Hg distribution indicates that the black alteration mainly consists of Hg.

**Figure 8:**
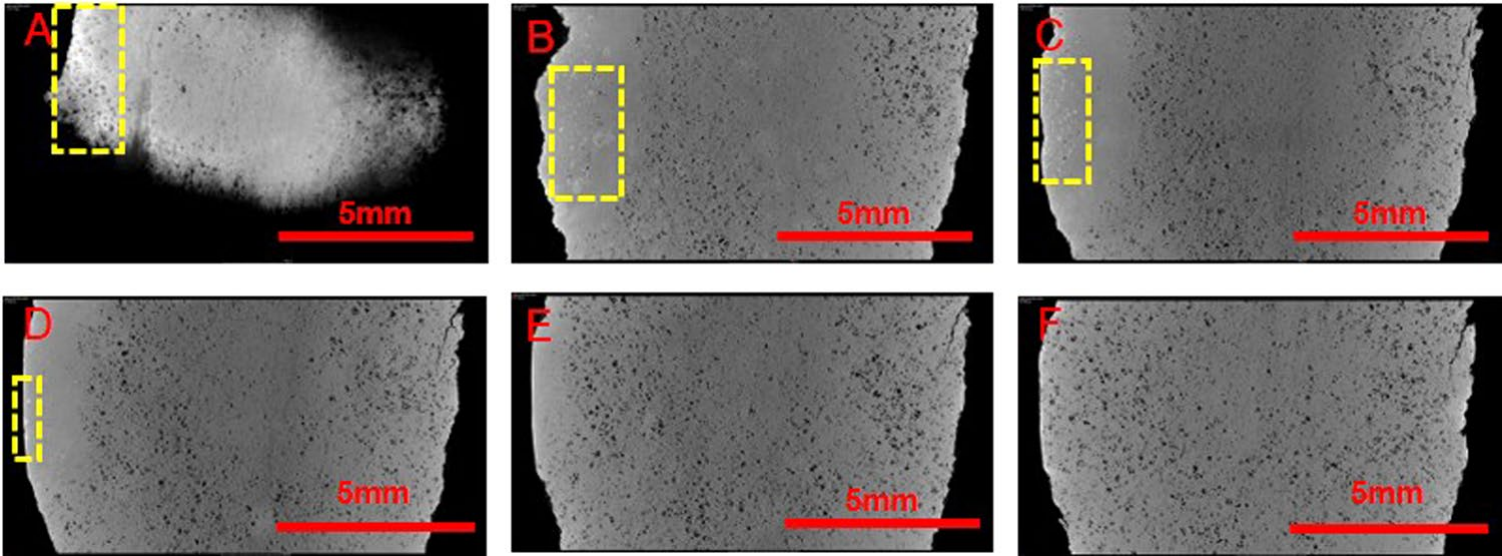
The CT images of G212-1. (**A**–**F**) Images are from one side to the other side in turn and the interval of each image is 250 μm. The light spots in the yellow imaginary area are mercury alteration.

As shown in the CT image (Fig. 8), a small quantity of high-density matter is distributed in the holes inside the structure of the jade samples. This high-density matter is mercury alteration since the Hg density is higher than that of the elements that comprise nephrite jades. The alteration permeates from the surface to the internal structure of the samples. The depth of its distribution is approximately 800 μm. This phenomenon also proves that the black material is a kind of alteration and not surface contamination.

Overall, we are convinced that the black alteration mainly consists of Hg and is distributed from the surface to the inner structure. This kind of black alteration is the mercury alteration recorded in ancient Chinese literature.

According to the heating phenomenon described for G21:2-1 and G21:2-2, we conclude that the studied ancient jade artifacts may have been heated with cinnabar (HgS) before being buried in Lizhou’ao Tomb. That is, heat-decomposed cinnabar provided the mercury that caused the mercury alteration on the samples. Cinnabar will be oxidized when the heating temperature exceeds 400 °C. According to the preceding data analysis, the two samples were heated over 800 °C. When the heating temperature surpassed 800 °C, the cinnabar decomposed and produced mercury vapor, generating the numerous holes observed in the jades. During the heating process, mercury adhered and entered the jades along holes and crevices with the help of heating and formed the mercury alteration. Mercury alteration is in fact the black mixture which mainly made of mercury oxide, and its black color is also related to the material.

As described in the older Chinese literature, the fire tradition (“Liaoji”), a ceremonial activity that involved burning oblations to receive blessings in ancient China, has lasted for over 7000 years since the Hongshan Culture. Jade was one of the most important oblations. Cinnabar was always placed under oblations considered the most auspicious^27^. In the fire tradition, jade and cinnabar provide the material base, and fire produces the high temperature. Therefore, the mercury alteration could have formed on jade oblations during the fire tradition ceremony. The tombs that contain ancient jade artifacts with mercury alteration may have belonged to high-class nobles.

According to the results of this study, all nine pieces of ancient jade from G21 in Lizhou’ao Tomb were heated in ancient times. This heating may imply the high status of the coffin occupant. Based to the heating phenomenon, it is reasonable to speculate the coffin occupant was involved in sacrificial activity, perhaps as a priest. Our study results also indicate that the Lizhou’ao area practiced the “Liaoji” custom during the Spring and Autumn Period. In addition, jade and cinnabar were heated during the fire tradition as part of the ceremony.

In conclusion, two ancient jade fragments with mercury alteration unearthed from Lizhou’ao Tomb in Jiangxi Province were studied using materials science analysis. The two jades are nephrite jades with obvious heating-related features. The color of their outside and inside sections has changed to yellowish-brown and white, respectively. The black color can be attributed to alteration on the artifacts. This alteration mainly consists of Hg and can be referred to as mercury alteration, as described in the older Chinese literature. The mercury alteration of the ancient jade artifacts is considered to have formed during the fire tradition ceremony. The occupant of G21 in Lizhou’ao Tomb may have been a high-class noble. The results of this study could be used in future studies on mercury alteration and jade culture in Chinese archeology.

## Conclusions

This paper describes the analysis of ancient jade artifacts with mercury alteration unearthed from Lizhou’ao Tomb in Jiangxi Province. The distribution of the ancient jades with mercury alteration was extended to Jiangxi Province. Ancient jades with mercury alteration could indicate substantial archeological significance. First, “Shuiyin qin” is the most scientific and explicit traditional proper name for mercury alteration in Chinese. Second, the appearance of mercury alteration on the studied artifacts indicates they may have been heated with cinnabar in the ancient times. Third, the jades with mercury alteration may have been used as oblations in ancient sacrificial activity. Fourth, the tombs that contain ancient jades with mercury alteration may have belonged to high-class nobles. Fifth, the mercury alteration reveals details regarding sacrificial offerings and methods in ancient sacrificial activities in specific ancient time periods. In addition, an integrated scientific method to study ancient jade artifacts with mercury alteration is established in this study. This nondestructive method could be widely used in the scientific and cultural study of ancient jades in Chinese archeology.

## Data Availability

The datasets generated and/or analyzed the current study are available from the corresponding author on reasonable request. All data generated or analyzed during this study are included in this published article.
